# Supplementary material for: Oncometabolite D-2-Hydroxyglutarate enhances gene silencing through inhibition of specific H3K36 histone demethylases
Source: eLife. 2017 Mar 27;6:e22451. doi: 10.7554/eLife.22451 (PMC5388528; doi:10.7554/eLife.22451)
Supplement: Supplementary file 1. — All strains were derived from W303 with the following genotype: can1-100 his3-11 leu2-3,11 lys2 TRP1 ADE2 ura3-1. DOI: http://dx.doi.org/10.7554/eLife.22451.015 [file elife-22451-supp1.docx]

**Supplementary Figures and Tables**

**Supplementary table 1** *Saccharomyces cerevisiae* strains used in this study. All strains were derived from W303 with the following genotype:  *can1-100 his3-11 leu2-3,11 lys2 TRP1 ADE2 ura3-1.*

| Name | Genotype |
| --- | --- |
| JRY10731 | *hmlα2∆::CRE ura3∆::pGPD:loxP:yEmRFP;tCYC1:kanMX:loxP:yEGFP:tADH1 IDP2-R132H* |
| JRY10732 | *hmlα2∆::CRE ura3∆::pGPD:loxP:yEmRFP;tCYC1:HygMX:loxP:yEGFP:tADH1 idp2*∆::*natmx* |
| JRY10733 | *hmlα2∆::CRE ura3∆::pGPD:loxP:yEmRFP;tCYC1:KanMX:loxP:yEGFP:tADH1 dld2∆::URA3^C. albicans^* |
| JRY10734 | *hmlα2∆::CRE ura3∆::pGPD:loxP:yEmRFP;tCYC1:kanMX:loxP:yEGFP:tADH1 dld2∆::URA3^C. albicans^ IDP2-R132H* |
| JRY10735 | *hmlα2∆::CRE ura3∆::pGPD:loxP:yEmRFP;tCYC1:kanMX:loxP:yEGFP:tADH1 dld2∆::URA3^C. albicans^ idp2∆::natmx* |
| JRY10736 | *hmlα2∆::CRE ura3∆::pGPD:loxP:yEmRFP;tCYC1:hygMX:loxP:yEGFP:tADH1 gis1∆::HIS3* |
| JRY10737 | *hmlα2∆::CRE ura3∆::pGPD:loxP:yEmRFP;tCYC1:hygMX:loxP:yEGFP:tADH1 rph1∆::HIS3* |
| JRY10738 | *hmlα2∆::CRE ura3∆::pGPD:loxP:yEmRFP;tCYC1:hygMX:loxP:yEGFP:tADH1 jhd1*∆*::kanmx* |
| JRY10739 | *hmlα2∆::CRE ura3∆::pGPD:loxP:yEmRFP;tCYC1:hygMX:loxP:yEGFP:tADH1 dld2∆::URA3 ^C. albicans^ IDP2-R132H idp1∆::URA3 ^C. albicans^* |
| JRY10740 | *hmlα2∆::CRE ura3∆::pGPD:loxP:yEmRFP;tCYC1:hygMX:loxP:yEGFP:tADH1 rph1∆::HIS3 jhd1*∆::KanMX |
| JRY10741 | *hmlα2∆::CRE ura3∆::pGPD:loxP:yEmRFP;tCYC1:hygMX:loxP:yEGFP:tADH1 dld2∆::URA3 ^C. albicans^ idp1∆::URA3 ^C. albicans^* |
| JRY10742 | *hmlα2∆::CRE ura3∆::pGPD:loxP:yEmRFP;tCYC1:hygMX:loxP:yEGFP:tADH1 rph1∆::HIS3 gis1∆::HIS3* |
| JRY10743 | *hmlα2∆::CRE ura3∆::pGPD:loxP:yEmRFP;tCYC1:hygMX:loxP:yEGFP:tADH1 rph1∆::HIS3 gis1∆::HIS3 jhd1∆::KanMX* |
| JRY10745 | *hmlα2∆::CRE ura3∆::pGPD:loxP:yEmRFP;tCYC1:hygMX:loxP:yEGFP:tADH1 gis1∆::HIS3 jhd1∆::KanMX* |
| JRY10746 | *hmlα2∆::CRE ura3∆::pGPD:loxP:yEmRFP;tCYC1:hygMX:loxP:yEGFP:tADH1 set2∆::URA3 ^C. albicans^* |
| JRY10747 | *hmlα2∆::CRE ura3∆::pGPD:loxP:yEmRFP;tCYC1:hygMX:loxP:yEGFP:tADH1 set2∆::URA3 ^C. albicans^ gis1∆::HIS3 rph1∆::HIS3* |
| JRY10748 | *ADE2/ADE2 lys2/lys2 TRP1/TRP1 hmlα2∆::CRE/hmlα2∆::CRE ura3∆::pGPD:loxP:yEmRFP;tCYC1:kanMX:loxP:yEGFP:tADH1/ura3∆::pGPD:loxP:yEmRFP;tCYC1:HygMX:loxP:yEGFP:tADH1 dld2∆::URA3^C. albicans^/dld2∆::URA3^C. albicans^ IDP2-R132H/IDP2-R132H* |
| JRY10749 | *ADE2/ADE2 lys2/lys2 TRP1/TRP1 hmlα2∆::CRE/hmlα2∆::CRE ura3∆::pGPD:loxP:yEmRFP;tCYC1:kanMX:loxP:yEGFP:tADH1/ura3∆::pGPD:loxP:yEmRFP;tCYC1:HygMX:loxP:yEGFP:tADH1 dld2∆::URA3^C. albicans^/dld2∆::URA3 ^C. albicans^* |
| JRY10750 | *ADE2/ADE2 lys2/lys2 TRP1/TRP1 hmlα2∆::CRE/hmlα2∆::CRE, ura3∆::pGPD:loxP:yEmRFP;tCYC1:hygMX:loxP:yEGFP:tADH1/ura3∆::pGPD:loxP:yEmRFP;tCYC1:HygMX:loxP:yEGFP:tADH1* |
| JRY10751 | *ADE2/ADE2 lys2/lys2 TRP1/TRP1 hmlα2∆::CRE/hmlα2∆::CRE ura3∆::pGPD:loxP:yEmRFP;tCYC1:kanMX:loxP:yEGFP:tADH1/ura3∆::pGPD:loxP:yEmRFP;tCYC1:kanMX: loxP:yEGFP:tADH1 dld2∆::URA3^C. albicans^/dld2∆::URA3 ^C. albicans^ IDP2-R132H/IDP2* |
| JRY10752 | *ADE2 lys2 TRP1 hmlα2∆::CRE ura3∆::pGPD:loxP:yEmRFP;tCYC1:hygMX:loxP:yEGFP:tADH1 dld3Δ::kanmx* |
| JRY10753 | *ADE2 lys2 TRP1 hmlα2∆::CRE ura3∆::pGPD:loxP:yEmRFP;tCYC1:hygMX:loxP:yEGFP:tADH1 dld3∆::kanmx dld2∆::URA3 ^C. albicans^* |
| JRY10754 | *ADE2 lys2 TRP1 hmlα2∆::CRE ura3∆::pGPD:loxP:yEmRFP;tCYC1:hygMX:loxP:yEGFP:tADH1 ecm5Δ::URA3 ^C. albicans^* |
| JRY10755 | *ADE2 lys2 TRP1 hmlα2∆::CRE ura3∆::pGPD:loxP:yEmRFP;tCYC1:HygMX:loxP:yEGFP:tADH1 jhd2Δ::Natmx* |
| JRY10756 | *ADE2 lys2 TRP1 hmlα2∆::CRE ura3∆::pGPD:loxP:yEmRFP;tCYC1:hygMX:loxP:yEGFP:tADH1 set1Δ::URA3 ^C. albicans^* |
| JRY10757 | *ADE2 lys2 TRP1 HML^+^ ura3∆::pGPD:loxP:yEmRFP;tCYC1:kanMX:loxP:yEGFP:tADH1* |
| JRY10758 | *ADE2 lys2 TRP1 HML^+^ ura3∆::pGPD:loxP:yEmRFP;tCYC1:kanMX:loxP:yEGFP:tADH1 dld2∆::URA3* |
| JRY10790 | *ADE2 lys2 TRP1 hmlα2∆::CRE ura3∆::pGPD:loxP:yEmRFP;tCYC1:hygMX:loxP:yEGFP:tADH1* |
| JRY10791 | *ADE2 lys2 TRP1 hmlα2∆::CRE ura3∆::pGPD:loxP:yEmRFP;tCYC1:hygMX:loxP:yEGFP:tADH1* |
| JRY10792 | *ADE2 lys2 TRP1 hmlα2∆::CRE ura3∆::pGPD:loxP:yEmRFP;tCYC1:hygMX:loxP:yEGFP:tADH1 dld2∆::URA3 ^C. albicans^ gis1∆::HIS3 rph1∆::HIS3* |
| JRY10793 | *ADE2 lys2 TRP1 hmlα2∆::CRE ura3∆::pGPD:loxP:yEmRFP;tCYC1:hygMX:loxP:yEGFP:tADH1 dld2∆::URA3 ^C. albicans^ IDP2-R132H rph1∆::HIS3 gis1∆::HIS3* |
| JRY10794 | *ADE2 lys2 TRP1 hmlα2∆::CRE ura3∆::pGPD:loxP:yEmRFP;tCYC1:kanMX:loxP:yEGFP:tADH1 dld2∆::URA3 ^C. albicans^ set2∆::URA3 ^C. albicans^* |
| JRY10795 | *ADE2 lys2 TRP1 hmlα2∆::CRE ura3∆::pGPD:loxP:yEmRFP;tCYC1:hygMX:loxP:yEGFP:tADH1 dld2∆::URA3 ^C. albicans^ IDP2-R132H set2∆::URA3 ^C. albicans^* |
